# EAC-Agent: A deep learning framework for multimodal emotion-aware conversational agent with contextual response generation

**DOI:** 10.1371/journal.pone.0346770

**Published:** 2026-04-17

**Authors:** Shahid Jamil, Tariq Ali, Asif Nawaz, Abdul Shahid

**Affiliations:** 1 University Institute of Information Technology, PMAS–Arid Agriculture University, Rawalpindi, Pakistan; 2 School of Business, South East Technological University, Waterford, Ireland; Hunan Normal University, CHINA

## Abstract

Conversational agents, commonly referred to as chatbots, have become an integral part of various important applications, such as customer support and virtual assistants in enterprise-level solutions. Despite their widespread use, current solutions predominantly rely on a single modality input, primarily text, which limits their ability to fully understand the correct emotions of users and respond accordingly. The increasing demand for multimodal inputs in conversation such as text, audio, and videos, highlights the need for a more comprehensive approach. Existing conversational agents face significant challenges in generating emotionally aware responses, as they lack the ability to effectively handle emotion embeddings, leading to limitations in emotional accuracy and contextual appropriateness. Focusing on these challenges, this research work leads to a novel multimodal approach which incorporates features from text, audio, and visual. The EAC-Agent proposed a sequence-to-sequence model with transformer, along with pre-trained embeddings such as GloVe. The self and cross-modal attention on text, audio and visual is used to generate a more emotionally intelligent response. EAC-Agent is validated through comparison with existing techniques on two benchmark datasets. The obtained results demonstrate superior performance in emotion classification and response generation. The proposed model achieves an accuracy of 76.27% on IEMOCAP and 67.57% on MELD for emotion recognition from multimodal user inputs. In addition, the emotion-aware response generation module shows clear improvements, with perplexity values of 39.01 and 42.30, BLEU scores of 0.31 and 0.30, and ROUGE-L scores of 0.45 and 0.44 on IEMOCAP and MELD, respectively. EAC-Agent demonstrates clear superiority over existing models and holds great promise for applications in customer service, healthcare, and other areas requiring empathetic and contextually appropriate interactions.

## Introduction

**Fig 1:**
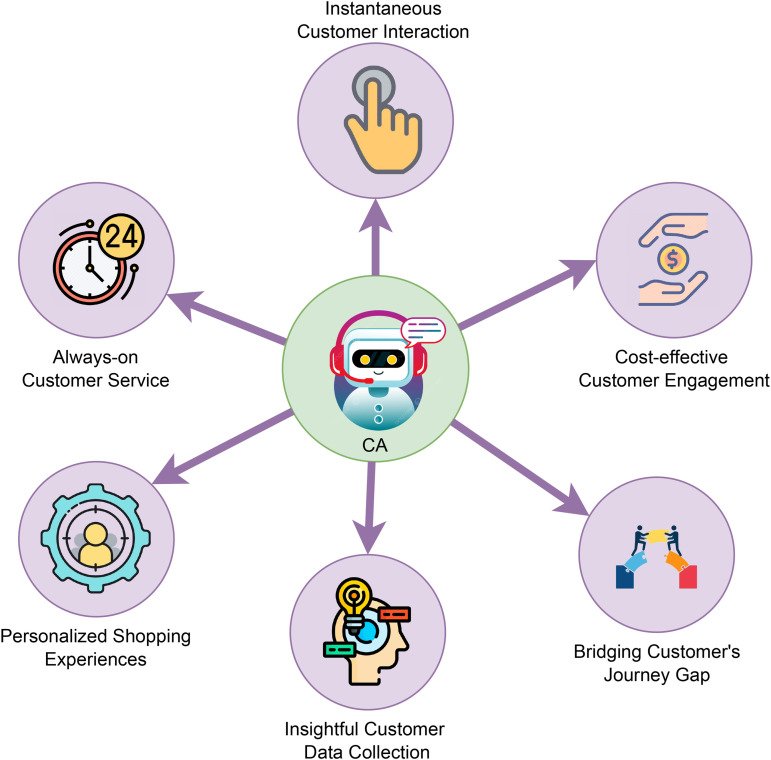
Advantages of conversational agents. The figure summarizes the key benefits of conversational agents in enhancing user interaction, personalization, and accessibility.

Chatbots, or conversational agents (CAs), are digital systems/programs that replicate human interaction through Natural Language Processing (NLP) [1]. Due to the interactivity and user-friendly designs of these technologies, many people prefer them over traditional static Frequently Asked Questions (FAQ) systems [2]. Over the years, CAs have become more important in numerous areas as a result of growing technological developments in AI. When you look at the different types of conversational systems, there are many benefits when compared to human agents: they can be available 24/7; they can engage with thousands of users simultaneously [3], and such agents provide a more tailored experience based on individual user data. As mentioned in Fig 1, these systems are being used in different domains, especially in healthcare, where they support patients in psychological treatments and provide medical information [4]. Similarly in business, they smoothly perform customer service, enhance the quality of service, and effectively minimize the cost by handling large numbers of customer queries [5]. In the field of education, CAs contribute to personalized tutoring and support interactive learning [6]. While talking about entertainment, they are again excellent in storytelling and gaming.

However, many existing conversational systems still struggle to accurately interpret the emotional context of user queries and respond in an empathetic manner, which can negatively affect user experience [7,8]. Initially designed for simple conversations and entertainment, such systems now encounter significant challenges related to emotional intelligence in responses. CAs may struggle to produce fully satisfactory responses when they lack awareness of a user’s emotional state. Whereas, if these systems are well aware of the emotional state of the users, they will be in a stronger position to satisfy users by generating empathetically more relevant responses [9]. For example, to improve communication, an effective conversational system should be able to detect when a user is angry and respond empathetically. Traditional text-based systems, however, struggle to accurately identify a user’s emotional state, as textual input alone cannot effectively capture emotional cues such as facial expressions and voice tone.

Multi-modal approaches are in a better position to understand user emotions, because they use text, audio, image and video modalities [10]. This, in turn, enables conversational systems to generate more empathetic responses. For example, images are best for detecting the facial expression of the user in a happy state. Similarly textual data are suitable for the disgust state rather from image. In conclusion, both happy and disgust emotion could be better identified when combined from these modalities [11]. Now users are more satisfied with CAs during conversation, and this is because of a multi-modal approach which is helping in better emotional detection [12]. CAs play especially more vital role in the situation when a user is in a frustrated state [13] and the best example is the field of customer service, where the emotion embedded response will normalize the depressed emotion of the customer. In light of an existing research study, 40–45% of queries from the users are other than normal state, which need empathetic response [14], so that the user will be feeling as s/he is talking to a human and showing sympathy.

In this work, emotion embeddings refer to dense latent vector representations learned by an emotion classifier that encode the user’s emotional state. These embeddings are not pre-softmax hidden activations or static class embeddings, but conditioning vectors derived from multimodal features. They are explicitly used to condition the response generation process, enabling the model to produce emotionally aligned responses.

While modern CAs utilize techniques such as sentiment analysis and machine learning to detect emotional content, there is still substantial room for improvement in their emotional intelligence [15]. Recently done research work is using deep learning techniques, enabling such agents to generate human-like responses [16]. Transformer-based models like Generative Pre-trained Transformer (GPT) and Bidirectional Encoder Representations from Transformers (BERT) excel in understanding and generating context-aware responses using attention mechanisms, building on earlier Seq2Seq models with attention mechanisms such as LSTM, GRU based architectures [17]. First, dialogue robots like DialoGPT are based on the transformer model created by Google (the Transformer). The transformer model used for dialogue robots has been trained using datasets that contain actual conversations among real human beings [18]. Second, memory networks like MANN have the ability to keep track of conversation history using an external source of memory, which allows the model to access and retrieve conversation history to generate contextually aware and accurate responses. In addition to these two technologies, research on memory networks and pointer networks has improved the capacity of CAs to incorporate context and increased the blankness and depth of their responses, as demonstrated by the memory networks.

Growing interest has emerged to develop multimodal affective conversational agents due to the development of new technologies. The model proposed introduces an innovative method of providing multimodal affective conversational agents by creating a fusion network that combines text, image, and audio features, to significantly improve the accuracy of emotion detection during engagement with users. The research work uses a semi-transformer-based model, which is further divided into four main components. The first component is required to compute the fixed-size vector text, visual and audio. Then positional embeddings are added to this vector and its purpose is to not miss the contextual information from each modality. For text, we used GloVe, for images we used Convolutional Neural Networks (CNN), and for audio we used Mel-Frequency Cepstral Coefficients (MFCC). The second component is about the fusion network, where we are using an early fusion approach by combining features from all the three modalities. The third component is the emotion classifier, to which the single fused feature vector is fed, and it generates the emotion embeddings. In the fourth and final component, both the generated embeddings and the user’s query are forwarded to the sequence to sequence with attention encoder to generate the emotion embedded response at the decoder side. All these components are helping the CAs to be more human-like.

The summarized contribution of this research work is as follows:

- This study addresses the limitations of unimodal emotion detection methods by proposing a multimodal approach that integrates text, visual, and audio features, offering a more comprehensive solution for emotion recognition during conversations.
- This study introduces a novel early fusion approach which integrates features extracted from text, visual and audio modalities which produce a higher accuracy of emotion detection.
- The proposed model embeds unique emotional features into generated responses, setting a new standard for multimodal emotion detection and response generation in conversational systems.
- The experimental evaluation indicates that the proposed model attains an overall accuracy of 76.27%, 67.57% and a weighted F1 score of 76.36%, 67.50% outperforming baseline models across benchmark IEMOCAP and MELD datasets respectively.

The remaining sections of the paper are organized as follows. In Related Work section, the literature in the relevant field is reviewed, while the Methodology section is all about the proposed methodology. In the Results and Discussion section, we reported the effectiveness of the proposed approach by setting up the experiments resulting in excellent results. Finally, the Conclusion section provides the summary of the research work and outlines future work.

## Related work

During the last several years, many researchers have contributed to the field of multimodal emotion-aware conversational agents, advancing techniques for emotion detection and response generation through the integration of text, visual, and audio modalities, the summary is described in Table 1.

**Table 1:** Comparative analysis of existing methods.

Ref	Methods	Dataset	Acc	Limitations
[19]	RNN, XGBoost, NB	CVAW3.0 (Text, Audio, Video)	66.5	FAQ-type dialogs only; no user perspective targeting
[20]	FFNN, RNN, GRUs	IEMOCAP (Text, Audio, Video)	65.7	Limited test samples; 20% abnormal dropout
[21]	Seq2Seq, HRED	PersonaChat, ConvAI2 (Text)	71.5	Binary emotion classification; limited response factors
[22]	CNNs, LSTMs, 3D-CNNs, Transformers	DailyDialog (Text, Audio, Video)	88.0	Cross-dataset generalizability issues; inconsistent evaluation metrics; limited real-world robustness
[23]	QuartzNet, BERT variants	MELD (Text, Audio, Video)	59.2	Dependence on human-generated transcripts
[24]	CNN, RNN/LSTM, attention mechanisms	CMU-MOSI (Text, Audio, Video)	73.2	Alignment and synchronization issues across modalities; class imbalance; lack of unified evaluation metrics
[25]	LSTM, Transformer, CNN	RAVDESS (Audio, Video)	80.0	Modality dominance issues; lack of large real-world corpora
[26]	CNN, RNN, hybrid fusion, attention models	RAVDESS (Audio, Video)	85.0	High computational cost; difficulties in real-time fusion; limited cross-domain generalizability

Acc = accuracy

Recent studies emphasize that relying on a single modality often limits a model’s ability to capture the full context of human interactions. Multimodal learning addresses this limitation by combining information from multiple sources, such as text, audio, and visual cues, allowing models to learn richer and more meaningful representations. By leveraging the complementary strengths of different modalities, deep learning–based fusion approaches have shown improved performance in tasks that require contextual understanding and affective awareness. These advances are particularly relevant for emotion-aware conversational agents, where integrating multimodal information can lead to more contextually appropriate and emotionally aligned response generation [27].

The emotion recognition system introduced by Ghosal et al. employs graph convolution neural network-based methods [28]. Their model uses both the intra-speaker (speaker’s own speech) and inter-speaker (the other person’s speech) sequential and contextual dependence of the utterance and uses that to classify the utterance as belonging to one of the previously labeled emotion classes. In the experiments performed, Ghosal et al. used only textual content and observed statistically significant improvements over the current leading classification methods. The authors observed that short utterances often contain multiple meanings and associations that can only be understood through the use of multimodal data to provide context for understanding (e.g., ‘ok. yes.’). When evaluated on the Multimodal Emotion Lines Dataset (MELD), the authors’ method produced an F1 measure of 58.10%.

Ho et al. proposed an approach to recognizing emotion in speech, which utilizes a combination of various modalities [29]. This approach uses both Recurrent Neural Network (RNN) and a self-to-multi-head attention mechanism. Firstly, they extract MFCC features from audio and use the BERT model for features from textual data. In the next phase, these features are fed into the RNN, where the self-attention layer is also activated. Features are fused using a multi-head attention mechanism to correctly predict the emotion from these modalities. The F1 score is computed separately for text and then from both text and speech on the MELD dataset. In text, it is 59.98%, whereas in multimodal it is 60.59%, showing an improvement by using multi-modal.

The study referred to in [30], examined the method employed within Deep Learning Algorithms to help provide an efficient means of determining emotions based on the combination of different types of data such as (Text, Video and Audio). The study identified that a text-based response is the most appropriate response for integrating emotion. There were two primary approaches considered in the study, the first being The Early Fusion Method and the second is The Late Fusion Method. It can be clearly demonstrated through the experimental results of this study that both of these methods were effective for allowing the system to utilize the emotion within the multimodal conversation to generate an effective emotionally embedded response. In addition, this approach improved the accuracy level of the system by 8% through the incorporation of these methods. The effectiveness of the response generated from an attention mechanism was used to measure the accuracy of the response.

Emotion detection in conversations has emerged as an increasingly important topic within the NLP community. The method that they used is their distinctive way of approach; it is simply using another source of information (audio) to help obtain and include additional contextual information regarding the person contributing to a conversation by way of an emotional detection model using GNNs (Graph Neural Networks) [31]. Thus, knowing “who” is speaking and “how” the person is talking plays a major factor in the overall effectiveness of the results produced by utilizing both of these sources. Because of the use of both forms of data, the results from their evaluation on the MELD dataset (Multimodal Emotion Detection) were noticeably above all other related models. But it’s also worth mentioning that their relatively low weighted measure (F1 Score of 55%) can likely be attributed to the dataset used in comparison to the complexity of the Emory NLP dataset.

The dynamics of multimodal sentiment and emotion analysis highlight the advances in method/techniques where there is more than just simple concatenation of inputs or static attention strategies; the first technique combines fuzzy cognition-based dynamic fusion networks (Fcdnet) with contextual relevance via fuzzy cognitive reasoning, allowing an adaptive balance of modality contributions and a more effective representation of the semantic and affective meaning across text, audio and visual input. The emphasis on cognitive/rationale value of each of the three modalities as well as their complementary relationship enhances feature interactions, therefore increasing the performance of multimodal sentiment benchmarks, indicating an exciting possibility for emotion-aware conversational systems that interpret nuanced emotional contexts via multiple data sources [32].

Several recent studies have focused on improving multimodal emotion recognition in conversations by combining information from text, audio, and visual signals. Ma et al. [33] propose a transformer-based model with self-distillation to strengthen cross-modal feature learning and achieve better results on the IEMOCAP and MELD datasets. Hu et al. [34] introduce MMGCN, which uses graph convolution networks to model interactions between speakers and different modalities. Mao et al. [35] present DialogueTRM, a hierarchical transformer model designed to capture emotional changes and long-range context in conversations. Similarly, Hu et al. [36] propose MM-DFN, which dynamically integrates multimodal features for more effective emotion recognition. All these approaches are evaluated on the widely used IEMOCAP and MELD datasets and represent important contributions to multimodal conversational emotion analysis.

In addition to widely used benchmarks such as IEMOCAP and MELD, prior studies have introduced a range of multimodal datasets to facilitate research in conversational emotion analysis and affective computing. The Multimodal Emotion Intensity and Sentiment Dataset (MEISD) goes beyond traditional emotion recognition methods because it explicitly models emotion intensity by combining textual, acoustic and visual representations [37]. CMU-MOSEI and CMU-MOSI Datasets are much larger scale multimodal annotated datasets that include both sentiment and emotion recognition, enabling analysis of multiple affective expression types based on spoken language, facial expression and vocal characteristics [24]. Other datasets such as Emory NLP [38], DailyDialog [39], and the OMG Emotion [40] help researchers learn and understand how emotional dynamics occur within the context of conversation or multimedia communication. Although there are many multimodal datasets currently available, IEMOCAP and MELD are still the most frequently utilized benchmarks for creating emotion responsive conversational agent systems, particularly when researchers compare their results against previously established baseline models [37].

Researching the literature regarding current research on emotion detection and response generation from conversational agents suggests that while many of these techniques have good accuracy and efficiency, there are also a number of limitations to these technologies as mentioned in Table 1. The implications of this study emphasize the critical need to develop advanced emotion-aware multimodal conversational agents that will allow for real-time human emotional recognition and response. Existing methods, including unimodal and some multimodal approaches, often struggle with challenges such as incomplete emotional context, modality misalignment, and the need for precise feature integration. The proposed model addresses these limitations by leveraging deep learning techniques, such as early fusion networks and attention mechanisms, to effectively combine text, audio, and visual modalities.

## Methodology

**Fig 2:**
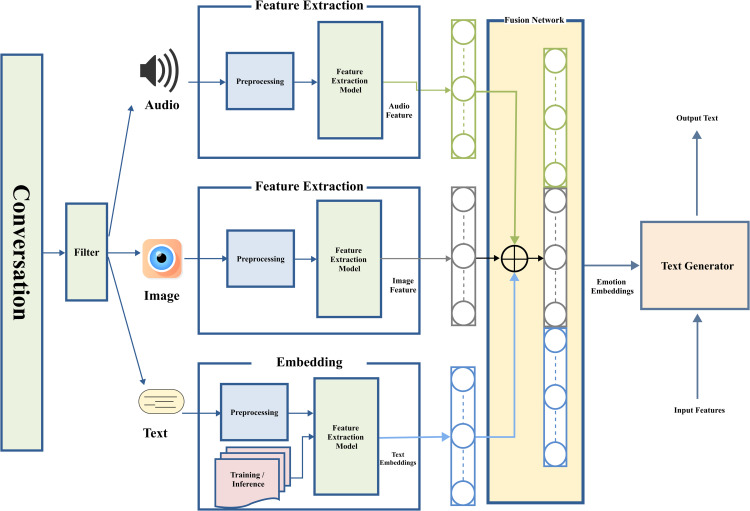
Proposed architecture of the EAC-Agent framework. The figure presents the overall workflow of the proposed system, including multimodal feature extraction, self- and cross-attention-based fusion, and emotion-aware response generation.

This section discusses the core methodology of EAC-Agent. Fig 2 shows the architecture and the complete workflow of the proposed research work, which is composed of four important components. Feature extraction is the very first phase in the sequence, then fusing the features from each modality. The next component is about emotion embeddings and the last one is responsible for text-based response generation.

### Preprocessing and feature extraction

In the preprocessing stage, we employed GloVe-based embedding strategy for text, Gaussian Mixture Model (GMM) for audio and Vision Transformer (ViT) for videos. On the other hand, feature extraction enables the identification and quantification of relevant patterns within the raw data, which in most cases are not immediately apparent for emotion detection. One of the basic purposes of extracting the key features from each modality is to reduce the data dimensionality. Text preprocessing is the key step to format the textual data in a way so that the feature extraction process will be performed more efficiently and, as a result, to improve the performance of the model. In text preprocessing, we first do tokenization, then cleaning, normalization, lemmatization and stemming. In the end, the padding and truncation step is performed. After that, the sequences will become a fixed length L:


$$\begin{array}{c} \text{pad\_sequence}=\left\{ \begin{array}{cc} \text{seq}+[\text{PAD},\ldots ], & \text{len(seq)}<L\\ \text{seq}[:L], & \text{len(seq)}\geq L \end{array}\right. \end{array}$$ (1)

Text embeddings transform the textual data into numerical vectors along with preserving the semantic relationships between words, which is ideal for the models to process. For embedding we use GloVe, which maps each word *w*_*i*_ to a dense vector $v_{w_{i}}\in {\mathbb{R}}^{d}$. The co-occurrence matrix *M*_*ij*_ counts the total number of word pairs in a context window:


$$M_{ij}=\sum_{k}I\left(w_{i}\text{ and }w_{j}\text{ co-occur in context of }w_{k}\right)$$ (2)

To extract features from audio, we perform audio preprocessing. In this process we first remove background noise from the audio by using spectral gating. After that, the audio signal ***x***_(*t*)_ is segmented into the overlapping frames of length N and hop of size H as:


$$x_{f}(t)=x(t)\cdot w(t-fH),\;f=0,1,2,\ldots ,N-1$$ (3)

where:

- *x*(*t*) is the original audio signal
- *w*(*t*) is the window function (e.g., Hann or Hamming window)
- *H* is the hop size (in samples)
- *f* is the frame index
- *N* is the total number of frames

For a signal of length *T* samples, the number of frames is:


$$F=\left\lfloor \frac{T-N}{H}\right\rfloor +1$$ (4)

For each frame, MFCCs are extracted with the help of Short-Time Fourier Transform (STFT):


$$X_{f}(k)=\sum_{t=0}^{N-1}x_{f}(t)e^{-j\frac{2\pi }{N}kt},\;k=0,1,\ldots ,N-1,$$ (5)

The resultant MFCC feature vector of frame *f* is $c_{f}=[c_{f}(0),c_{f}(1),\ldots ,c_{f}(K-1)]$. The GMM is then used for feature modelling:


$$p(c_{f})=\sum_{g=1}^{G}w_{g}𝒩(c_{f};\mu_{g},\Sigma_{g}),$$ (6)

where *w*_*g*_ is the weight, $\mu_{g}$ is a vector of mean values and $\Sigma_{g}$ is the covariance matrix of size *g* × *g* having the *g*^*th*^ Gaussian component. To represent the audio signal as a fixed length embedding, the mean and variance of the GMM components are computed and concatenated. The final audio embedding ***e*** is given by:


$$e=[\mu_{1},\mu_{2},\ldots ,\mu_{G},\sigma_{1}^{2},\sigma_{2}^{2},\ldots ,\sigma_{G}^{2}],$$ (7)

Where $\mu_{g}$ and $\sigma_{g}^{2}$ are the mean and variance of the *g*^*th*^ Gaussian component. This results in a fixed length representation that captures the underlying structure of the audio signal.

Video preprocessing begins with frame extraction, where a video sequence $V=\{I_{1},I_{2},\ldots ,I_{T}\}$ is divided into *T* frames, each of size *H* × *W* × *C*. Each frame *I*_*t*_ is split into non-overlapping patches of size *P* × *P*, resulting in $N_{patches}=\left(\frac{H}{P}\right)\left(\frac{W}{P}\right)$ patches per frame. These patches are flattened into vectors $p_{t,i}\in {\mathbb{R}}^{P^{2}C}$ for the *i*^*th*^ patch in frame *I*_*t*_:


$$p_{t,i}=flatten\left(I_{t}\left[{patch}_{i}\right]\right)$$ (8)

The patches are then linearly embedded into fixed-length vectors $z_{t,i}\in {\mathbb{R}}^{D}$ using a learnable weight matrix $W_{embed}\in {\mathbb{R}}^{D\times (P^{2}C)}$ and bias $b_{embed}\in {\mathbb{R}}^{D}$:


$$z_{t,i}=W_{embed}\cdot p_{t,i}+b_{embed}$$ (9)

Positional encodings ***e***_*i*_, generated using sinusoidal functions, are added to retain spatial information:


$$e_{i}^{\left(2k\right)}=\sin\left(\frac{i}{10000^{2k/D}}\right),$$ (10)

$$e_{i}^{\left(2k+1\right)}=\cos\left(\frac{i}{10000^{2k/D}}\right)$$ (11)

Resulting in the final input $z_{t,i}^{\prime }=z_{t,i}+e_{i}$ for the Vision Transformer (ViT).

The embedded patches $z_{t,i}^{\prime }$ are processed by the Vision Transformer, which consists of multi-head self-attention and feed-forward layers. The self-attention mechanism computes attention weights using queries ***Q***, keys ***K***, and values ***V***:


$$Attention(Q,K,V)=softmax\left(\frac{QK^{⊤}}{\sqrt{d_{k}}}\right)V$$ (12)

The output is passed through a feed-forward network with ReLU activation:


$$h_{i}=ReLU\left(W_{2}\cdot ReLU\left(W_{1}\cdot z_{i}^{\prime }+b_{1}\right)+b_{2}\right)$$ (13)

The sequence of patch embeddings is aggregated into a single video-level representation $e_{video}$ by using the class token $z_{cls}^{final}$:


$$e_{video}=\frac{1}{N_{patches}}\sum_{i=1}^{N_{patches}}z_{i}$$ (14)

The final video embedding $e_{video}\in {\mathbb{R}}^{D}$ captures both spatial and temporal characteristics, enabling downstream tasks such as classification and action recognition.

**Fig 3:**
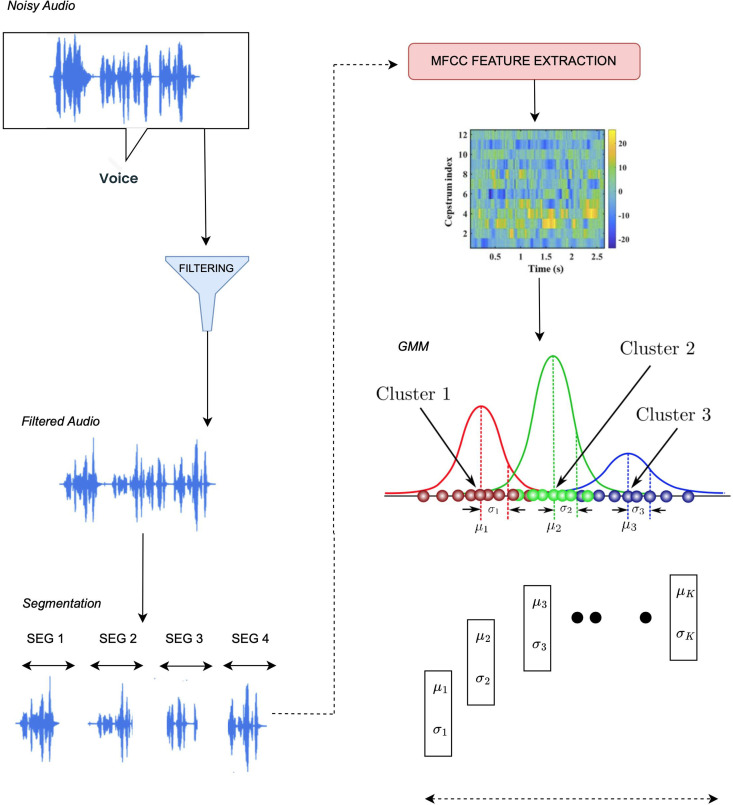
Audio feature extraction process. The figure illustrates the steps involved in noise reduction, MFCC computation, and statistical modeling for generating acoustic representations.

Fig 3 presents the process adopted for deriving audio features in the proposed framework. The input speech signal is initially preprocessed to suppress background noise and remove unwanted distortions, yielding a refined audio signal. From this processed signal, MFCCs are computed to capture relevant spectral patterns associated with human speech perception. These coefficients are subsequently summarized using simple statistical measures, such as their average and dispersion, to obtain a fixed-length representation that can be effectively utilized for emotion-aware modeling.

**Fig 4:**
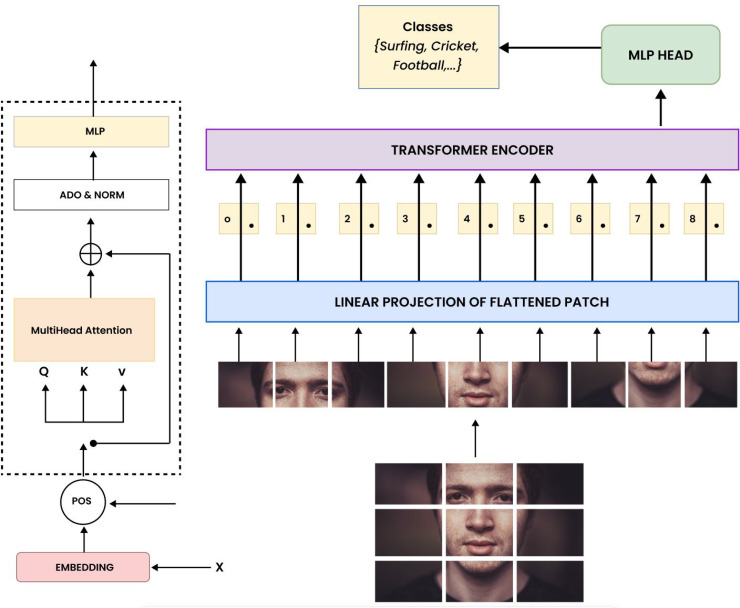
Video feature extraction using Vision Transformer (ViT). The figure illustrates the process of patch extraction, embedding, and self-attention-based feature learning for visual representation.

Fig 4 outlines the procedure used to extract visual features within the proposed framework. Each input image is first segmented into a set of uniform patches, which are subsequently converted into vector representations and augmented with positional information to retain spatial order. These patch embeddings are then fed into a Transformer encoder, where self-attention mechanisms and feed-forward layers model interactions across different regions of the image. The final output is produced through an MLP-based projection, yielding compact visual representations suitable for subsequent analysis or prediction tasks.

### Feature fusion using self and cross-attention

Given the embeddings from each modality (i.e., text, audio and visual) our aim is to fuse information both temporally and across modalities. Let us denote these as:


$$𝒯=\{t_{1},t_{2},\ldots ,t_{N}\}$$ (15)

$$𝒜=\{a_{1},a_{2},\ldots ,a_{N}\}$$ (16)

$$𝒱=\{v_{1},v_{2},\ldots ,v_{N}\}$$ (17)

where $t_{i},a_{i},v_{i}\in {\mathbb{R}}^{d}$ represent the feature embeddings at the *i*^*th*^ utterance for the text, audio, and video modalities, respectively.

### Self-attention within modalities

Self-attention is applied independently within each modality across its temporal utterance sequence. This mechanism captures intra-modal temporal dependencies:


$${𝒯}^{\prime }=SelfAttn(𝒯)$$ (18)

$${𝒜}^{\prime }=SelfAttn(𝒜)$$ (19)

$${𝒱}^{\prime }=SelfAttn(𝒱)$$ (20)

For each modality ℳ∈{𝒯,𝒜,𝒱}, the self-attention mechanism computes:


$$Attention(Q,K,V)=softmax\left(\frac{QK^{⊤}}{\sqrt{d_{k}}}\right)V$$ (21)

where the query, key, and value matrices are computed as:


$$Q=ℳW^{Q}$$ (22)

$$K=ℳW^{K}$$ (23)

$$V=ℳW^{V}$$ (24)

with $W^{Q},W^{K},W^{V}\in {\mathbb{R}}^{d\times d_{k}}$ being learnable parameters.

### Cross-modal attention

Self-attention mechanisms are effective at modeling temporal relationships within individual modalities; however, they are insufficient for capturing interactions between different modalities. To overcome this, we introduce a directional cross-modal attention strategy that allows each modality to incorporate relevant cues from the others in a selective manner. Let ***t***_*i*_, ***a***_*i*_, and $v_{i}\in {\mathbb{R}}^{d}$ represent the self-attended embeddings of text, audio, and visual modalities, respectively, at utterance index *i*. The cross-modal attention process is defined directionally as $\left(m_{1}\leftarrow m_{2}\right)$, indicating that modality *m*_1_ receives contextual information from modality *m*_2_, which serves as the source. For each ordered modality pair $\left(m_{1}\leftarrow m_{2}\right)$, the cross-attended representation $c_{i}^{\left(m_{1}\leftarrow m_{2}\right)}$ is computed using scaled dot-product attention:


$$c_{i}^{\left(m_{1}\leftarrow m_{2}\right)}=softmax\left(\frac{Q_{i}^{\left(m_{1}\right)}(K_{i}^{\left(m_{2}\right)}{)}^{⊤}}{\sqrt{d_{k}}}\right)V_{i}^{\left(m_{2}\right)},$$ (25)

where $Q_{i}^{\left(m_{1}\right)}\in {\mathbb{R}}^{1\times d_{k}}$ denotes the query vector generated from the target modality *m*_1_, $K_{i}^{\left(m_{2}\right)}\in {\mathbb{R}}^{1\times d_{k}}$ denotes the key vector generated from the source modality *m*_2_, $V_{i}^{\left(m_{2}\right)}\in {\mathbb{R}}^{1\times d_{k}}$ denotes the value vector from the source modality, and *d*_*k*_ is the dimensionality of the attention subspace used for scaling. The query, key, and value vectors are obtained through linear projections as follows:


$$Q_{i}^{\left(m_{1}\right)}=m_{1,i}W_{Q}^{\left(m_{1}\leftarrow m_{2}\right)},$$ (26)

$$K_{i}^{\left(m_{2}\right)}=m_{2,i}W_{K}^{\left(m_{1}\leftarrow m_{2}\right)},$$ (27)

$$V_{i}^{\left(m_{2}\right)}=m_{2,i}W_{V}^{\left(m_{1}\leftarrow m_{2}\right)},$$ (28)

where $m_{1,i}\in {\mathbb{R}}^{1\times d}$ and $m_{2,i}\in {\mathbb{R}}^{1\times d}$ represent the target and source modality embeddings at utterance *i*, respectively, and $W_{Q}^{\left(m_{1}\leftarrow m_{2}\right)}$, $W_{K}^{\left(m_{1}\leftarrow m_{2}\right)}$, and $W_{V}^{\left(m_{1}\leftarrow m_{2}\right)}\in {\mathbb{R}}^{d\times d_{k}}$ are learnable projection matrices specific to the modality pair.

### Text-guided cross-modal attention

To integrate acoustic and visual emotional cues into textual representations, the text modality attends to both audio and visual modalities:


$$c_{i}^{\left(T\leftarrow A\right)}=softmax\left(\frac{(t_{i}W_{Q}^{\left(T\leftarrow A\right)})(a_{i}W_{K}^{\left(T\leftarrow A\right)}{)}^{⊤}}{\sqrt{d_{k}}}\right)(a_{i}W_{V}^{\left(T\leftarrow A\right)}),$$ (29)

$$c_{i}^{\left(T\leftarrow V\right)}=softmax\left(\frac{(t_{i}W_{Q}^{\left(T\leftarrow V\right)})(v_{i}W_{K}^{\left(T\leftarrow V\right)}{)}^{⊤}}{\sqrt{d_{k}}}\right)(v_{i}W_{V}^{\left(T\leftarrow V\right)}),$$ (30)

where $c_{i}^{\left(T\leftarrow A\right)}$ and $c_{i}^{\left(T\leftarrow V\right)}$ denote the cross-attended textual representations conditioned on audio and visual information, respectively.

### Audio-guided cross-modal attention

To allow acoustic representations to be guided by semantic and visual context, the audio modality attends to text and visual modalities:


$$c_{i}^{\left(A\leftarrow T\right)}=softmax\left(\frac{(a_{i}W_{Q}^{\left(A\leftarrow T\right)})(t_{i}W_{K}^{\left(A\leftarrow T\right)}{)}^{⊤}}{\sqrt{d_{k}}}\right)(t_{i}W_{V}^{\left(A\leftarrow T\right)}),$$ (31)

$$c_{i}^{\left(A\leftarrow V\right)}=softmax\left(\frac{(a_{i}W_{Q}^{\left(A\leftarrow V\right)})(v_{i}W_{K}^{\left(A\leftarrow V\right)}{)}^{⊤}}{\sqrt{d_{k}}}\right)(v_{i}W_{V}^{\left(A\leftarrow V\right)}),$$ (32)

where $c_{i}^{\left(A\leftarrow T\right)}$ and $c_{i}^{\left(A\leftarrow V\right)}$ represent audio features enhanced by textual and visual information, respectively.

### Visual-guided cross-modal attention

To align visual emotional expressions with linguistic and acoustic signals, the visual modality attends to text and audio modalities:


$$c_{i}^{\left(V\leftarrow T\right)}=softmax\left(\frac{(v_{i}W_{Q}^{\left(V\leftarrow T\right)})(t_{i}W_{K}^{\left(V\leftarrow T\right)}{)}^{⊤}}{\sqrt{d_{k}}}\right)(t_{i}W_{V}^{\left(V\leftarrow T\right)}),$$ (33)

$$c_{i}^{\left(V\leftarrow A\right)}=softmax\left(\frac{(v_{i}W_{Q}^{\left(V\leftarrow A\right)})(a_{i}W_{K}^{\left(V\leftarrow A\right)}{)}^{⊤}}{\sqrt{d_{k}}}\right)(a_{i}W_{V}^{\left(V\leftarrow A\right)}),$$ (34)

where $c_{i}^{\left(V\leftarrow T\right)}$ and $c_{i}^{\left(V\leftarrow A\right)}$ denote visual representations augmented with textual and acoustic cues, respectively.

In the proposed design, each cross-modal attention unit is equipped with projection layers tailored to specific modality pairs, allowing the model to learn detailed alignment relationships between heterogeneous inputs. By structuring the attention in a directional manner, information from one modality is incorporated into another in a controlled and selective way. The outputs of this cross-modal interaction are then combined with the corresponding self-attended representations, producing a unified feature representation that supports multimodal emotion comprehension and response generation.

### Fused representation

The final fused representation ***F***_*i*_ at each time step *i* is obtained by concatenation:


$$F_{i}=Concat\left(\begin{array}{c} t_{i}^{\prime },a_{i}^{\prime },v_{i}^{\prime },\\ c_{i}^{\left(𝒯\leftarrow 𝒜\right)},c_{i}^{\left(𝒯\leftarrow 𝒱\right)},\\ c_{i}^{\left(𝒜\leftarrow 𝒯\right)},c_{i}^{\left(𝒜\leftarrow 𝒱\right)},\\ c_{i}^{\left(𝒱\leftarrow 𝒯\right)},c_{i}^{\left(𝒱\leftarrow 𝒜\right)} \end{array}\right)$$ (35)

This representation captures both temporal dependencies within modalities and cross-modal interactions, enabling comprehensive multimodal emotion understanding.

**Fig 5:**
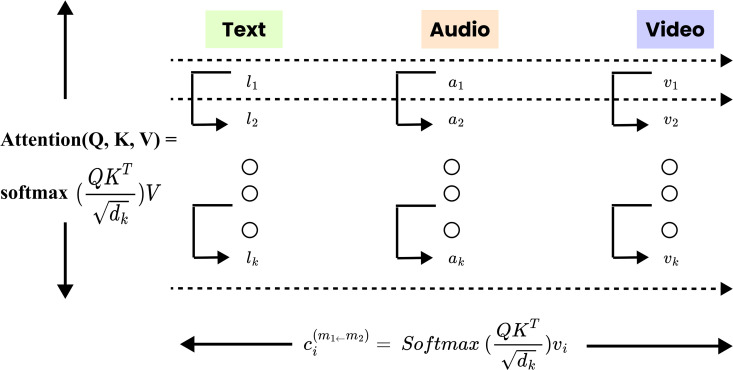
Self and cross attention mechanism. The figure illustrates the interaction between self-attention and cross-attention for multimodal feature fusion.

Fig 5 describes how attention mechanisms are used to combine information from multiple modalities. For each individual modality (text, audio, or video) self-attention is applied to learn internal relationships among its constituent elements. In contrast, cross-attention operates across modalities, allowing features from one stream to be informed by representations from the others. Through this coordinated interaction, the model is able to fuse complementary cues from different sources, resulting in representations that better reflect both contextual dependencies and emotional nuances.

### Response generation

Following the multi-modal fusion stage, we obtain a sequence of fused representations $\left\{F_{1},F_{2},\ldots ,F_{N}\right\}$, where each $F_{i}\in {\mathbb{R}}^{d_{f}}$ captures both temporal and cross-modal dependencies at utterance *i*. The response generation module utilizes this enriched multi-modal context to generate emotionally aware, contextually appropriate responses.

We employ a Transformer decoder to auto regressively generate the target response $Y=\{y_{1},y_{2},\ldots ,y_{M}\}$, where $y_{j}\in 𝒱$ is the token at position *j*, and 𝒱 denotes the vocabulary. The decoder attends to the fused representations $\{F_{i}{\}}_{i=1}^{N}$ via encoder-decoder cross-attention:


$$z_{j}=CrossAttn\left(h_{j},\{F_{i}{\}}_{i=1}^{N}\right)=softmax\left(\frac{Q_{j}K^{⊤}}{\sqrt{d_{k}}}\right)V$$ (36)

where:


$$Q_{j}=h_{j}W^{Q}$$ (37)

$$K=FW^{K}$$ (38)

$$V=FW^{V}$$ (39)

with $W^{Q},W^{K},W^{V}\in {\mathbb{R}}^{d_{f}\times d_{k}}$ being learnable parameters. Here, CrossAttn(·) denotes a standard scaled dot-product attention mechanism that aligns the decoder hidden state with the fused multimodal context. Specifically, the decoder hidden representation $h_{j}\in {\mathbb{R}}^{1\times d_{f}}$ at generation step *j* is projected to a query vector $Q_{j}\in {\mathbb{R}}^{1\times d_{k}}$, while the fused multimodal representations $F=[F_{1},F_{2},\ldots ,F_{N}{]}^{⊤}\in {\mathbb{R}}^{N\times d_{f}}$ are projected to key and value matrices $K,V\in {\mathbb{R}}^{N\times d_{k}}$. The attention weights are computed by measuring the similarity between the query and each key vector, normalised using the softmax function, and are subsequently used to compute a weighted sum over the value vectors. This operation enables the decoder to selectively attend to the most relevant multimodal emotional context when generating each response token.

The token probability distribution is computed as:


$$P(y_{j}|y_{<j},F)=softmax\left(W_{o}z_{j}+b_{o}\right)$$ (40)

where $W_{o}\in {\mathbb{R}}^{|𝒱|\times d_{k}}$ and $b_{o}\in {\mathbb{R}}^{\left|𝒱\right|}$.

### Training objective

The model is trained to minimize the cross-entropy loss:


$${ℒ}_{rs}=-\sum_{j=1}^{M}\log P\left(y_{j}^{true}|y_{<j},F\right)$$ (41)

This architecture ensures responses are conditioned on both:

- The sequence of prior tokens (*y*_<*j*_)
- The temporally-aware, modality-aligned emotional context (***F***)

## Results and discussion

In this section we look into the results obtained from the performed experiments to measure the performance of the proposed model. The evaluations proved that the proposed model is performing better than the baseline models.

### Dataset

There are several datasets available, as already discussed in the literature review. We have chosen two well-known multi-modal datasets, Multimodal Emotion Lines Dataset (MELD) and Interactive Emotional Dyadic Motion Capture (IEMOCAP), since they are widely used benchmark datasets in literature [23,33–36]. Table 2 describes the complete details of each dataset along with its availability source. The first dataset is IEMOCAP, including text, audio and video modalities of 12 hours duration. The dataset includes conversations between ten different actors. Each conversation is annotated with one of the six emotions used in the dataset. The second dataset is the MELD, which is also a multimodal dataset containing text, audio and video data. This dataset includes 13,000 utterances from the Friends TV series and 1,400 dialogues. Each utterance from the MELD dataset is annotated with one of the seven emotions used in the dataset. Further information about the pre-processed multimodal features used in this study are provided in supporting study **S1 File** (IEMOCAP) and **S2 File** (MELD).

**Table 2:** Multimodal datasets and their description.

Name	Description	Emotions	Web link
IEMOCAP	Contains multimodal recordings of scripted and improvised conversations, including text, audio, and video transcriptions.	Happy, sad, neutral, angry, excited, frustrated	Request
MELD	Contains dialogues from movie scripts with emotional annotations, including text, audio, and video data.	Neutral, surprise, fear, sadness, joy, disgust, anger	GitHub

Table 3 presents the complete statistical picture of the two datasets in a brief way by classifying the total number of conversations, and utterances in each. The datasets are split into training and testing sets in 80:20 ratio. The utterances in IEMOCAP are organized into five sessions, with the first four sessions designated for training and the final session reserved for testing. Each utterance is assigned one of the emotion labels.

**Table 3:** Statistics of the datasets.

Dataset	No. of conversations		No. of utterances	
	**Train + Val**	**Test**	**Train + Val**	**Test**
IEMOCAP	120	31	5810	1623
MELD	1153	280	11098	2610

### Performance evaluation measure

The four common standards are mostly used to check how effective the model will be. Hence, we also used these four measures to claim that the proposed model is an effective one. The following equations are used for precision, recall, F1 score and accuracy:

We employ standard classification metrics to evaluate model performance:


$$\text{Precision}=\frac{TP}{TP+FP}$$ (42)

$$\text{Recall}=\frac{TP}{TP+FN}$$ (43)

$$F_{1}\text{-score}=2\times \left(\frac{\text{Precision}\times \text{Recall}}{\text{Precision}+\text{Recall}}\right)$$ (44)

$$\text{Accuracy}=\frac{TP+TN}{TP+TN+FP+FN}$$ (45)

To measure classifier effectiveness, we use:


$$\text{ROC}(i)=\frac{CP(i|\text{positive})}{CP(i|\text{negative})}$$ (46)

where:

- *CP*(*i*|positive) is the cumulative proportion of positive class samples
- *CP*(*i*|negative) is the cumulative proportion of negative class samples
- *i* represents the classification threshold

### Baseline methods

The study compares the proposed model with the following baseline approaches:

- **Baseline 1: SDT** [33] presents a transformer-based model with self-distillation, enabling knowledge transfer from both hard and soft labels to each modality. The architecture employs:
  $${ℒ}_{\text{SDT}}=\alpha {ℒ}_{\text{hard}}+(1-\alpha ){ℒ}_{\text{soft}}$$
  where α balances the loss terms.
- **Baseline 2: MMGCN** [34] constructs a conversation graph 𝒢=(𝒱,ℰ) with multimodal nodes and edges, applying graph convolution:
  $$H^{\left(l+1\right)}=\sigma \left({\tilde{D}}^{-\frac{1}{2}}\tilde{A}{\tilde{D}}^{-\frac{1}{2}}H^{\left(l\right)}W^{\left(l\right)}\right)$$
  where $\tilde{A}$ is the adjacency matrix with self-loops.
- **Baseline 3: DialogueTRM** [35] uses hierarchical transformers with multi-grained fusion:
  $$F_{i}=\sum_{m\in \{T,A,V\}}\beta_{m}\cdot {\text{TRM}}_{m}(X_{m})$$
  where $\beta_{m}$ are learnable modality weights.
- **Baseline 4: MM-DFN** [36] implements dynamic fusion via:
  $$g_{t}=\sigma (W_{g}[f_{t}^{𝒯};f_{t}^{𝒜};f_{t}^{𝒱}])$$
  where ***g***_*t*_ is the gating vector at time *t*.

## Results

We compared our proposed fusion model with four different fusion models to claim that it is leading all four. Tables 4 and 5 represent the performance of baseline methods and EAC-Agent on IEMOCAP and MELD datasets, respectively. Results show that our model performs better as compared to the state-of-the-art on overall performance both in terms of accuracy and weighted F1 (w-F1) score on both IEMOCAP and MELD Datasets. The rest of the three models are below SDT. In both Tables 4 and 5, the last row reports the overall accuracy and w-F1.

**Table 4:** Results on IEMOCAP dataset.

Emotions	MMGCN		DialogueTRM		MM-DFN		SDT		EAC-Agent	
	Acc	F1	Acc	F1	Acc	F1	Acc	F1	Acc	F1
Happy	50.00	56.25	72.22	62.84	57.64	52.87	72.71	**66.19**	**72.22**	65.75
Sad	78.78	81.43	**85.71**	83.33	84.49	**86.07**	79.51	81.84	83.27	83.27
Neutral	71.35	67.57	69.27	68.12	76.04	71.66	76.33	74.62	**81.82**	**79.00**
Anger	68.24	66.29	**79.41**	66.67	70.59	65.04	71.88	**69.73**	73.53	68.45
Excited	75.92	76.82	67.22	75.00	73.24	75.26	76.79	80.17	**79.60**	**82.20**
Frustrated	65.09	64.92	57.22	63.28	55.91	62.19	**67.14**	**68.68**	66.93	72.50
Overall	69.62	69.61	69.87	69.93	69.87	69.91	73.95	74.08	**76.27**	**76.36**

Acc = accuracy; F1 = F1-score; EAC-Agent = Proposed Model. Best results are shown in bold.

**Table 5:** Results on MELD dataset.

Emotions	MMGCN		DialogueTRM		MM-DFN		SDT		EAC-Agent	
	Acc	F1	Acc	F1	Acc	F1	Acc	F1	Acc	F1
Neutral	81.53	79.20	83.44	79.54	**83.52**	79.65	83.22	80.19	82.96	**80.40**
Surprise	58.36	57.75	54.45	57.09	**63.35**	58.17	61.28	59.07	61.56	**59.20**
Fear	8.00	13.79	24.00	**27.91**	**32.00**	26.67	13.80	17.88	14.09	17.70
Sadness	31.73	39.40	33.17	40.95	26.44	35.71	**34.90**	**43.69**	34.13	43.10
Joy	**69.90**	63.43	60.45	62.79	63.68	**64.89**	63.24	64.29	64.43	64.40
Disgust	20.59	24.56	22.06	28.04	19.12	24.76	22.65	28.78	**25.04**	**30.30**
Anger	52.17	53.49	**58.26**	53.96	49.28	52.15	56.93	**54.33**	55.65	53.50
Overall	66.40	65.21	66.70	65.76	66.55	65.48	67.55	66.60	**67.57**	**67.50**

Acc = accuracy; F1 = F1-score; EAC-Agent = Proposed Model. Best results are shown in bold.

**Fig 6:**
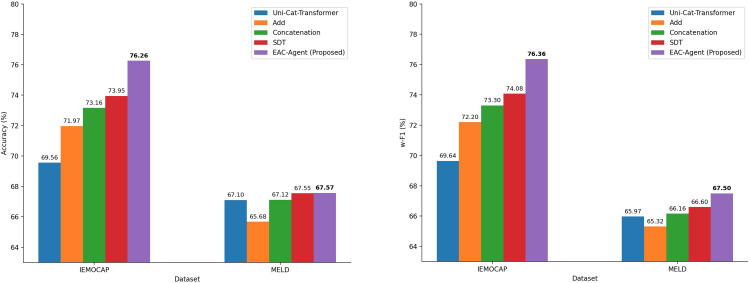
Performance comparison of different fusion methods on the two datasets. The figure compares the classification performance of various fusion strategies on the IEMOCAP and MELD datasets.

Fig 6 shows that the proposed fusion strategy significantly outperforms other fusion methods. The results suggest that direct fusion via Add or Concatenation is suboptimal. The proposed method enhances performance by first filtering irrelevant information at the unimodal level, then dynamically assigning weights across modalities at the multimodal level, resulting in more effective multimodal representation fusion.

**Fig 7:**
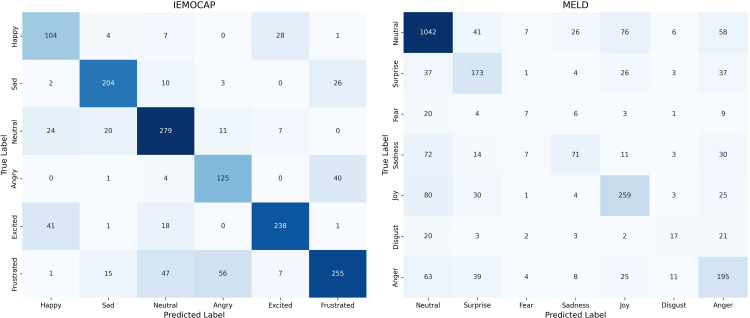
Confusion matrices for emotion classification. The figure illustrates the classification performance of the proposed model on the IEMOCAP and MELD datasets.

The confusion matrices in Fig 7 show that the proposed model performs well on both the IEMOCAP and MELD datasets, especially for common emotions such as Neutral, Sad, and Joy. Some confusion can be seen between closely related emotions, such as Happy and Excited or Angry and Frustrated in IEMOCAP, as well as between Surprise and Neutral in MELD, which reflects the similarity in their emotional expressions.

To evaluate the generated response and also the embedded emotion of the proposed model, we use Perplexity, BLEU-4 and ROUGE-L score as described in Tables 6 and 7. It can be observed that image and audio modalities are included in these evaluations, although they are not commonly used with BLEU and ROUGE metrics. Since BLEU-4 and ROUGE-L are text-based evaluation metrics, audio and image modalities are included as input conditioning signals to guide the generation of textual responses. These modalities provide complementary emotional and contextual cues, such as vocal tone and facial expressions, that influence response generation but are not directly evaluated. The reported scores therefore reflect the quality of the generated text under different conditioning settings, allowing analysis of how each modality contributes to response coherence and emotional alignment.

**Table 6:** Model performance across different test sets.

Dataset	Test Set	Perplexity(↓)	BLEU-4(↑)	ROUGE-L(↑)
IEMOCAP	TestSet-1	39.01	0.29	0.43
	TestSet-2	37.50	0.31	0.45
MELD	TestSet-1	42.30	0.27	0.41
	TestSet-2	41.15	0.30	0.44

↓ indicates lower values are better, ↑ indicates higher values are better.

**Table 7:** Results of EAC-Agent on Perplexity, BLEU, and ROUGE scores across modalities.

Dataset	Modality	Perplexity(↓)	BLEU-4(↑)	ROUGE-L(↑)
IEMOCAP	Text	34.85	0.28	0.42
	Audio	46.32	0.22	0.39
	Image	40.71	0.24	0.40
MELD	Text	31.64	0.32	0.45
	Audio	43.18	0.27	0.41
	Image	37.92	0.29	0.43

### Ablation study

To understand the importance and the contribution of different modalities in correct emotion detection, we performed tests by applying different ablation settings.

The ablation analyses of both the IEMOCAP and MELD datasets appear in Table 8, which shows how important the contributions of each modality are relative to one another in terms of how individually or in combination they contribute toward an overall improvement in performance. The text-only modality performs best among all of the modalities on both datasets, attaining an accuracy of 66.42% for IEMOCAP and 66.82% for MELD. The results suggest that the linguistic content of text provides the most reliable and strongest signal for emotion detection, as text contains explicit semantic and contextual information, whereas the acoustic modalities contain many ambiguous acoustic cues such as pitch and intensity that convey emotional intensity and do not convey the speaker’s intention. Similarly, the performance of the visual-only modality is the weakest among all modalities, particularly for IEMOCAP with accuracy at 42.45%, which is likely due to the variability of facial expressions from speaker to speaker as well as occluded features, changed poses during the recording, and/or misaligned visual frames; thus, clearly indicating that visual information alone should not be relied upon to infer emotional intent.

**Table 8:** Ablation study on IEMOCAP and MELD.

Model/Modality	IEMOCAP		MELD	
	Acc (%)	w-F1 (%)	Acc (%)	w-F1 (%)
**EAC-Agent (Proposed)**	**76.27**	**76.36**	**67.57**	**67.50**
*Single-modality results*				
Text only	66.42	66.58	66.82	65.52
Audio only	59.89	59.12	48.23	42.45
Visual only	42.45	43.56	48.89	33.45
*Modality combination results*				
Text + Audio	74.45	74.12	69.78	68.90
Text + Visual	71.23	70.89	69.89	68.12
Audio + Visual	66.56	65.78	49.12	43.19

Acc = accuracy; w-F1 = weighted F1-score.

When modalities are combined, a consistent performance improvement is observed. The text+audio configuration provides the most substantial gain among all bimodal combinations, achieving 74.45% accuracy on IEMOCAP and 69.78% on MELD. This improvement highlights the complementary relationship between semantic information from text and prosodic cues from audio, which together enable more robust emotion discrimination. The text+visual combination also improves performance compared to single modalities, although its gains are slightly lower than text+audio, suggesting that visual cues are beneficial but less stable than acoustic features. The audio+visual combination yields comparatively limited improvement and performs substantially worse than text-inclusive configurations, particularly on MELD (49.12% accuracy). This observation underscores the importance of linguistic grounding in emotion understanding, as non-textual modalities alone lack sufficient contextual information to accurately infer emotional intent. Finally, the proposed EAC-Agent, which integrates all three modalities, achieves the best overall performance across both datasets, with accuracies of 76.27% on IEMOCAP and 67.57% on MELD. This confirms that jointly modeling text, audio, and visual information allows the system to capture complementary emotional cues more effectively than any individual or partial modality combination.

## Conclusion

In this proposed work, we introduced a modern CA which allows users to express their emotions in many ways: like text, audio and visual. A novel approach is proposed to fuse these multiple modalities in a more effective way by using deep learning techniques. As a result, the CA is in a better position to engage the user by generating an empathetic response, which means the user feels that he is talking to a sincere companion. The experiments show that the users are more satisfied after taking a session with the CA, and, on the other hand, they stayed longer than the one with the traditional CA. The proposed work offers an ideal framework for developing an intelligent CA, which will be the best contribution to NLP as well as Human-Computer Interaction (HCI). In the future this research could be the baseline to enhance it across different languages, and also include the cultural context of the region. Moreover, this research can play a vital role in domains like customer service and mental health.

## Supporting information

S1 File iemocap multimodal features.pkl. Pre-processed multimodal feature dataset derived from the IEMOCAP corpus, and is publicly available at https://figshare.com/ndownloader/files/61784017.
(DOCX)

S2 File meld multimodal features.pkl. Pre-processed multimodal feature dataset derived from the MELD corpus, and is publicly available at https://figshare.com/ndownloader/files/61784020.
(DOCX)
