# Supplementary material for: EAC-Agent: A deep learning framework for multimodal emotion-aware conversational agent with contextual response generation
Source: PLoS One. 2026 Apr 17;21(4):e0346770. doi: 10.1371/journal.pone.0346770 (PMC13089751; doi:10.1371/journal.pone.0346770)
Supplement: S1 File — Pre-processed multimodal feature dataset derived from the IEMOCAP corpus, and is publicly available at https://figshare.com/ndownloader/files/61784017. (DOCX) [file pone.0346770.s001.docx]

This file serves as a placeholder because the actual dataset exceeds the 100 MB upload limit of Editorial Manager. The dataset is publicly available at [<https://figshare.com/ndownloader/files/61784017>] and is described in the manuscript’s Supporting Information captions.
